# Mechanisms involved in *Escherichia coli* and *Serratia marcescens* removal during activated sludge wastewater treatment

**DOI:** 10.1002/mbo3.196

**Published:** 2014-07-16

**Authors:** Maite Orruño, Idoia Garaizabal, Zaloa Bravo, Claudia Parada, Isabel Barcina, Inés Arana

**Affiliations:** Department of Immunology, Microbiology and Parasitology, Faculty of Science and Technology, University of Basque Country UPV/EHUBarrio Sarriena s/n, E-48940, Leioa, Spain

**Keywords:** Activated sludge, *gfp*-tagged strains, indicators, wastewater treatment

## Abstract

Wastewater treatment reduces environmental contamination by removing gross solids and mitigating the effects of pollution. Treatment also reduces the number of indicator organisms and pathogens. In this work, the fates of two coliform bacteria, *Escherichia coli* and *Serratia marcescens,* were analyzed in an activated sludge process to determine the main mechanisms involved in the reduction of pathogenic microorganisms during wastewater treatment. These bacteria, modified to express green fluorescent protein, were inoculated in an activated sludge unit and in batch systems containing wastewater. The results suggested that, among the different biological factors implied in bacterial removal, bacterivorous protozoa play a key role. Moreover, a representative number of bacteria persisted in the system as free-living or embedded cells, but their distribution into liquid or solid fractions varied depending on the bacterium tested, questioning the real value of bacterial indicators for the control of wastewater treatment process. Additionally, viable but nonculturable cells constituted an important part of the bacterial population adhered to solid fractions, what can be derived from the competition relationships with native bacteria, present in high densities in this environment. These facts, taken together, emphasize the need for reliable quantitative and qualitative analysis tools for the evaluation of pathogenic microbial composition in sludge, which could represent an undefined risk to public health and ecosystem functions when considering its recycling.

## Introduction

The development of wastewater treatment was driven by the need to reduce environmental contamination brought about by the uncontrolled discharge of polluted effluents into rivers and streams. The principal goals of wastewater treatment processes are the elimination of gross solids and mitigation of the effects of pollution via reduction of the readily assimilable organic fraction of the effluents and removal of slowly biodegradable organic matter, nitrogen, and phosphorus. Treatment also reduces the number of microorganisms, including indicator organisms and pathogens. A fecal coliform removal of around two logarithmic units (Garcia-Armisen and Servais 2007) or higher (Wéry et al. 2008; Muela et al. 2011) has been reported for wastewater treatment plants (WWTP) using an activated sludge process. Such a reduction is attributed both to abiotic factors, such as adsorption to flocs and settling (Reinthaler et al. 2003; Godfree and Farrell 2005), and to biological treatment, specifically to the complex interrelationships that are established between the microbial populations present in the wastewater (e.g., predation by bacterivorous protozoa, bacteriophage lysis and competition from wastewater bacteria) (Atlas and Bartha 1997). The reduction of bacteria can be also due to spontaneous cell death. These abiotic and biotic factors are vital steps in the activated sludge process and directly influence the quality of the effluent. However, the behavior of microorganisms during wastewater treatment might not be equally affected by the processes involved in bacterial elimination. Thus, bacteria could differentially interact with flocs and escape sedimentation (Zita and Hermansson 1997; Olofsson et al. 1998), and bacterial removal via the natural microbiota can vary depending on several factors (Wanjugi and Harwood 2013). Prey discrimination by bacterivorous protozoa based on cell size, cell wall composition or other factors has been described (Thurman et al. 2010). Banning et al. (2003) established that the availability of nutrients enhanced competition for nutrients or antagonisms between *Escherichia coli* (fecal bacteria) and the indigenous microbial population, resulting in reduced survival potential for *E. coli*.

Moreover, in the last few years, some studies (van Frankenhuyzen et al. 2011; Rowan 2011) have emphasized the potential risk of the induction of viable but nonculturable bacteria (VBNC) during wastewater treatment. The presence of these VBNC cells would lead to the underestimation of viable pathogen numbers in samples with the use of plating methods (van Frankenhuyzen et al. 2011), and the possible regrowth of VBNC pathogens after land application could create potential human health concerns (Sidhu and Toze 2009). At present, the information available about the significance and impact of VBNC organisms is still insufficient; therefore, as Rowan (2011) has suggested, critical data must be acquired to convert VBNC-phase potential pathogens from unknown entities to known and defined hazards.

Pathogen risk indicators, such as fecal coliforms and *E. coli,* are usually used to evaluate water quality and wastewater treatment efficiency, given the expense and technical challenges required for the evaluation of pathogenic microorganisms (Harwood et al. 2005; Werker et al. 2007; Ishii and Sadowsky 2008). These bacterial indicators are assumed to be indirect measures of the probability that pathogens are present (Bonadonna et al. 2002). However, absence of an indicator does not guarantee the absence of a pathogen, as the inactivation of pathogens depends on their nature and the type of treatment process (Bonadonna et al. 2002).

Knowledge of the fate of indicator microorganisms during treatment implies the ability to distinguish them from the rest of wastewater microorganisms. The use of genes encoding fluorescent proteins as markers for tracking and visualizing bacteria in environmental samples has been reported as an alternative for studies of complex ecosystems (Arana et al. 2003), including wastewater (Eberl et al. 1997; Olofsson et al. 1998). However, the release of large quantities of tagged microorganisms into a wastewater system involves a risk that renders this type of study unfeasible. To solve this problem, laboratory scale-activated sludge units (ASUs) can be used. ASUs have been demonstrated to be very accurate models of large-scale activated sludge plants and provide good and repeatable results (McClure et al. 1989).

In the present study, the objective was to evaluate the fate of bacteria during activated sludge wastewater treatment by measuring effective elimination, discharge in the effluent or accumulation in the sludge. For this, the following mechanisms involved in these processes were evaluated: interaction with wastewater microbiota, adsorption and settling and the generation of nonculturable populations. We used *E. coli* and *Serratia marcescens* bacteria modified to express the green fluorescent protein (GFP) protein as a biological model. The fate of these *gfp*-tagged populations was analyzed in two different assays: batch and ASU experiments.

## Materials and Methods

### Crispijana and laboratory scale WWTP

Samples were taken from the Crispijana WWTP (Vitoria-Gasteiz, Spain), which was previously characterized by Muela et al. (2011).

A laboratory scale-activated sludge unit simulating Crispijana WWTP working conditions was used in this study. The ASU consisted of a 12 L aeration tank and a 7 L secondary clarifier with a tapered bottom that enabled accumulation of the settled sludge. This sludge was partially returned to the aeration tank by a peristaltic pump, while the clarified effluent was removed via a liquid overflow to a drum. The ASU, filled with secondary effluent and sludge from the Crispijana WWTP, was fed with primary effluent at a rate of 1.1 L h^−1^, with a constant hydraulic retention time (HRT) of 10.9 h. The mixed liquor in the aeration tank was aerated and mixed with an air pump and an air-diffuser. The ASU experiments were carried out at room temperature.

To verify that both the Crispijana and laboratory scale WWTPs operated under similar conditions, primary and secondary effluent samples from both treatment plants were analyzed. Samples were collected monthly over a year from the Crispijana WWTP, and six experiments were performed using the ASU. Biological oxygen demand (BOD_5_), suspended solids (SS), and pH were measured (APHA, AWWA and WPCF 2005). The International Organization for Standardization (ISO) standard methods were used for the detection of heterotrophic bacteria (ISO 6222:^1999^) *E. coli* (ISO 9308-3:^1998^) and intestinal enterococci (ISO 7899-1:^1998^). The microplates used for detection of *E. coli*, and intestinal enterococci were supplied by Bio-Rad (Marnes-La-Coquette, France). The reduction efficiency of wastewater secondary treatment was determined (Muela et al. 2011) for the physicochemical (BOD and SS) and microbiological parameters.

### Bacterial strains

For this study, *E. coli* ABC_*gfp*_ (Orruño et al. 2013), resistant to gentamicin (10 *μ*g mL^−1^) and chloramphenicol (30 *μ*g mL^−1^), and *S. marcescens* pGEN222 (see below), resistant to ampicillin (128 *μ*g mL^−1^), were used. Another two green fluorescent *E. coli* lab strains, *E. coli* pEGFPlucTet (Lehtinen et al. 2004), which is resistant to tetracycline (5 *μ*g mL^−1^), and *E. coli* DH5*α* pGEN222 (kindly provided by V. de Lorenzo, CNB, Madrid), which is resistant to ampicillin (128 *μ*g mL^−1^), were also tested.

Transformation of *S. marcescens* strain Spanish Type Culture Collection (CECT) 159 with the plasmid DNA pGEN222 (Galen et al. 1999) was performed using a standard electroporation technique (Green and Sambrook 2012). Transformants were selected by ampicillin resistance (128 *μ*g mL^−1^) and green fluorescence emission.

Strains were stored at ^−^80°C using the Microbank™ bacterial preservation system (Pro-Lab Diagnostics, Richmond Hill, Canada).

Cell surface hydrophobicity (CSH) was measured as the ability of the strains to adhere to a hydrocarbon (xylene; Sigma-Aldrich, Madrid, Spain) as described by Rosenberg et al. (1980). The ability for biofilm formation (ABF) was measured as described by O'Toole and Kolter (1998). *Escherichia coli* ABC_*gfp*_ (CSH = 40.83 ± 7.66, ABF = 0.519 ± 0.066; *n* = 5), and *S. marcescens* pGEN222 (CSH = 31.57 ± 14.99, ABF = 0.325 ± 0.0129; *n* = 5) were both considered moderately hydrophobic (Basson et al. 2008) and strongly and moderately adherent, respectively (Stepanovic et al. 2000).

These derivative strains (*E. coli* ABC_*gfp*_ and *S. marcescens* pGEN222) exhibited the same growth rates and CSH and ABF values as their nontagged parental strains (data not shown).

### Fate of *E. coli* and *S. marcescens* during wastewater treatment: experimental designs

For batch experiments, wastewater samples taken from primary effluent were used. These wastewater samples were autoclaved (121°C, 15 min), were filtered through a 0.22-*μ*m pore size membrane filters (GVWP filters, Millipore, Madrid, Spain) or were used unprocessed and distributed in flasks. These flasks, containing 250 mL of wastewater, were inoculated with the *E. coli* or *S. marcescens gfp*-tagged strains at a final density of 10^7^–10^8^ bacteria mL^−1^. Bacterial suspensions were prepared as previously described by Arana et al. (2003). The inoculated flasks were incubated in the dark at 20°C with shaking (120 rpm). Triplicate subsamples were periodically collected for microbial counts. The similar survival of parental and derivative strains and the maintenance of fluorescence emission in tagged cells were checked in autoclaved wastewater (data not shown).

For ASU experiments, water samples were periodically taken to verify that working conditions were maintained during the experiments. Once the steady state was reached, samples were collected from the aeration tank (aqueous fraction and flocs), secondary clarifier (aqueous fraction and sludge), and secondary effluent. Before inoculation with *gfp*-tagged cells, wild *E. coli* cells were specifically enumerated by catalyzed reporter deposition fluorescence in situ hybridization (CARD-FISH) (see below) and by the most probable number (MPN) method (ISO 9308-3:^1998^,b^1998^). The ASU was fed continuously over 4 h with primary effluent inoculated with ∼1–4 × 10^6^ *gfp*-tagged *E. coli* or *S. marcescens* mL^−1^.

Furthermore, dry weights and the volumes of flocs and sludge were determined.

### Microbiological parameters for batch experiments

The total direct counts of bacteria (TDC) was directly enumerated using the standard acridine orange direct procedure described by Hobbie et al. (1977). For the enumeration of total *gfp*-tagged bacteria, unstained subsamples (TGFP) were examined directly by epifluorescence microscopy (Arana et al. 2003). In experiments with autoclaved wastewater, no differences were detected between green fluorescence emission and acridine orange-based counts. Thus, in experiments with unprocessed wastewater samples, the total number of wastewater bacteria was estimated as TDC minus TGFP (Arana et al. 2003).

Colony-forming units (CFU) were enumerated by the spread plate method on nutrient agar (Panreac, Castellar del Vallès, Barcelona, Spain). Plates were incubated for 24 h at 37°C, and green fluorescent colonies (CGFP) were enumerated under illumination at 360 nm (TL-K 40W/10-R UV-A; Philips, Madrid, Spain).

For ciliate protist enumeration, within 24 h after sampling, aliquots were stained with DAPI (4′,6-diamidino-2-phenylindole) (Porter and Feig 1980), filtered through 3-*μ*m pore size filters and observed with an epifluorescence microscope. In these experiments, from microbial counts obtained for unprocessed wastewater samples, the ratios *E. coli* or *S. marcescens*/ciliate protist and heterotrophic bacteria/ciliate protist were estimated.

Bacteriophages were quantified with the double agar layer technique following the ISO 10705-2 standard method (ISO 2000) using *E. coli* CN-13 (ATCC strain 700609) as the host bacteria for enumeration of somatic coliphages. The concentrations were expressed in plaque-forming units (PFU) mL^−1^.

### Microbiological parameters for ASU experiments

To extract bacteria from flocs and sludge, the samples were left to settle for 15 min. Then, bacteria from the settled phase were extracted by sonication (Sonics Vibra Cell, Newtown, CT) and recovered according to Orruño et al. (2013). The extraction efficiency of this method and the maintenance of cellular integrity during extraction were previously tested.

When the ASU was inoculated with *gfp*-tagged populations, direct enumeration was carried out in unstained subsamples by epifluorescence microscopy. In these experiments, the percentage of *E. coli* or *S. marcescens* cells remaining in the different fractions (aqueous and solid fractions and secondary effluent) was calculated in relation to the total cells introduced until the time of each sampling.

To establish the fate of fecal bacteria during the activated sludge treatment, the wild *E. coli* population, selected as an indicator bacterium, was enumerated by CARD-FISH. In ASU experiments with uninoculated wastewater, CARD-FISH was carried out according to Pernthaler et al. (2002) using the Colinsitu oligonucleotide probe (biomers.net) (Regnault et al. 2000; Nielsen et al. 2009). To avoid problems related to background fluorescence or nonspecific fluorescence deposits, recommendations described by Nielsen et al. (2009) were followed. Simultaneously, culturable wild *E. coli* cells were determined by the MPN method (ISO 9308-3:^1998^a,b^1998^).

### Statistical analysis

Statistical analysis was carried out with the StatView program (Abacus Concepts Inc., Berkeley, CA). All the results from the batch and ASU experiments presented below are the means of at least three experiments, and the coefficients of variation between replicates were <12%. Differences between means were assessed by analysis of variance (ANOVA). *P* ≤ 0.05 was considered significant.

## Results

### Comparison between the Crispijana and laboratory scale WWTPs

**Figure 1:**
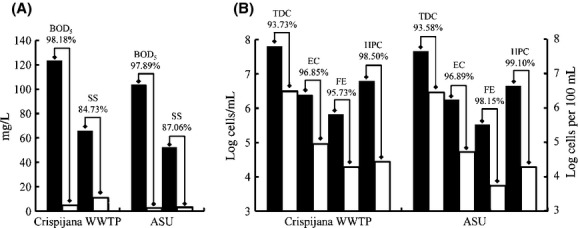
Reduction efficiencies of physicochemical (A) and microbiological (B) parameters during wastewater secondary treatment in the Crispijana wastewater treatment plant (WWTP) and in the laboratory-activated sludge unit (ASU). BOD_5_, biological oxygen demand; SS, suspended solids; TDC, total direct counts of bacteria (cells mL^−1^); EC, *Escherichia coli* (cells 100 mL^−1^); FE, intestinal enterococci (cells 100 mL^−1^); HPC, bacterial plate counts at 36°C (cells mL^−1^) in primary effluent (closed bars) and secondary effluent (open bars).

To assess that the ASU operated under conditions that simulated the Crispijana WWTP, different physicochemical and microbiological parameters were measured in primary and secondary effluents. The results obtained for each parameter as well as their reduction efficiencies are shown in Figure1. In terms of the physicochemical parameters (Fig.1A), few differences (*P* > 0.05) were observed between the WWTPs. In both of them, as expected, the BOD_5_ was highly reduced (>97% of reduction efficiency). The biological evaluations also demonstrated similar results for the Crispijana and laboratory scale WWTPs (Fig.1B), with 93–99% reduction efficiencies for all the parameters studied.

Moreover, the HRT for the Crispijana WWTP was estimated as 9.1 h (data provided by AQUALIA Gestión Integral del Agua S.A.-Lagunketa [UTE Crispijana]), slightly lower than the value obtained for the ASU (10.9 h).

### Fate of *E. coli* and *S. marcescens* in batch experiments

**Figure 2:**
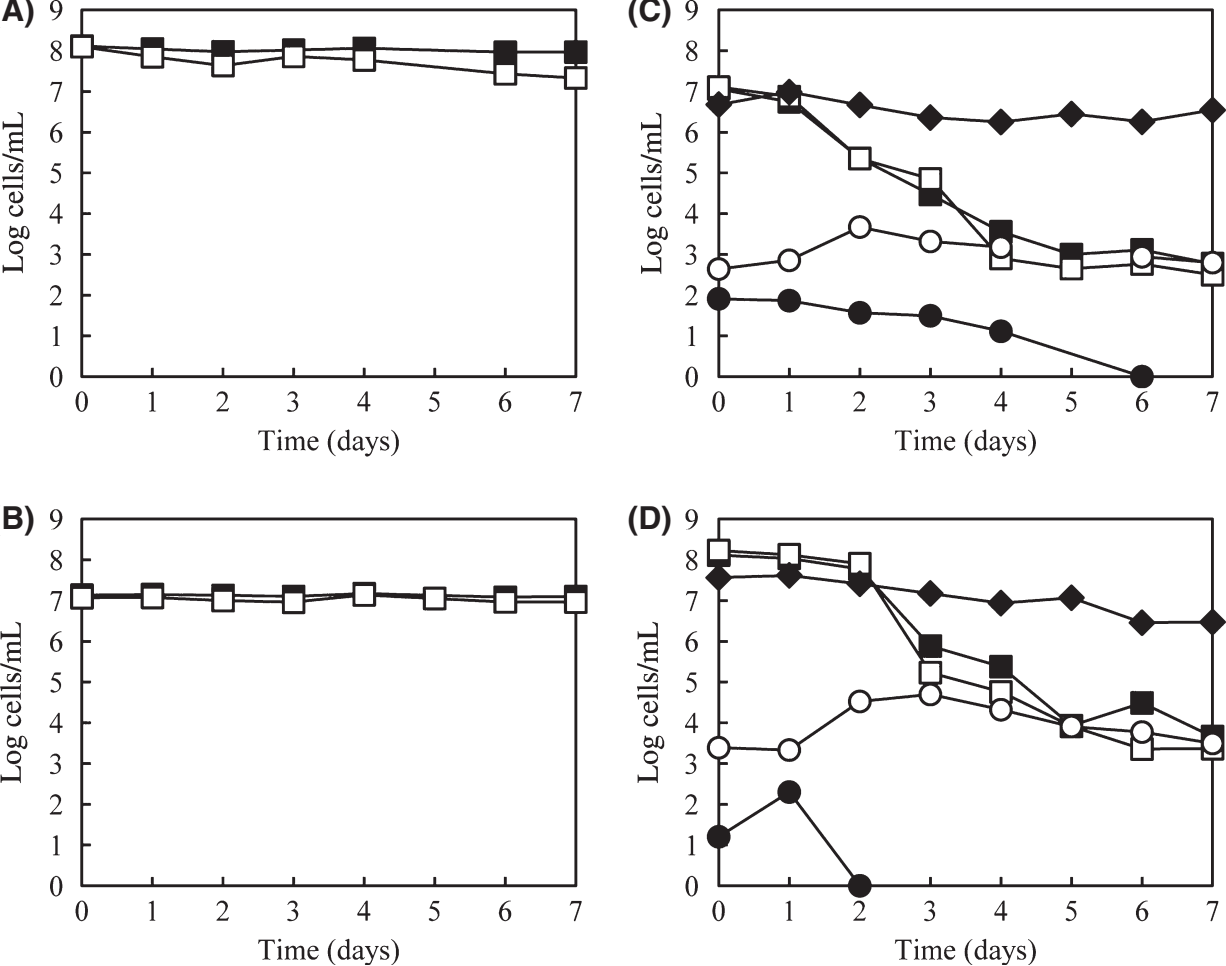
Survival of *Escherichia coli* ABC*gfp* (A and C) and *Serratia marcescens* pGEN222 (B and D) in autoclaved (A and B) and unprocessed (C and D) settled wastewater samples. Total direct counts (closed symbols) and culturable counts (open symbols) of *gfp*-tagged bacteria (■, □) and total direct counts of wastewater bacteria (**♦**). Number of ciliated protozoa (○) and number of bacteriophages (●). Data are averages of three experiments.

The different *gfp*-tagged *E. coli* strains tested exhibited similar behavior in batch experiments (data not shown). In Figure2, the results obtained for *E. coli* ABC_*gfp*_, and *S. marcescens* pGEN222 are presented. After 7 days in autoclaved wastewater in the absence of microbial wastewater populations, the *S. marcescens* (Fig.2B) population maintained cellular integrity and culturability, while *E. coli* (Fig.2A) cells suffered a slight decrease in culturability (∼0.8 log).

In an experiment designed to study the effect of bacteriophages, with wastewater filtered through 0.22-*μ*m pores, the evolution of total and culturable tagged *E. coli* subpopulations did not differ from the subpopulations observed in autoclaved wastewater (data not shown). In these samples, somatic coliphage populations were present, but they did not increase in number and were undetectable after 5 days of study (data not shown).

In the presence of the total microbiota (unprocessed wastewater), total and culturable counts of *gfp*-tagged strains demonstrated similar profiles, therefore, VBNC bacteria were not detected. There was a clear decreasing trend for *E. coli* (Fig.2C) and *S. marcescens* (Fig.2D); for both, their counts were ∼4 logs lower at the end of the study. However, the *E. coli* population began to decrease earlier than the *S. marcescens* population.

In these experiments, the total heterotrophic bacterial community did not undergo important changes during the study period but rather just a slight reduction when *S. marcescens* cells were introduced. Therefore, there was a clear distinction between the behavior of the wastewater bacteria, which in a large part maintained their density, and the introduced bacterial populations, which were drastically reduced in number. The ratio *E. coli* or *S. marcescens*/ciliate protist diminished 4 orders along the incubation time. However, the ratio heterotrophic bacteria/ciliate protist remained constant or decreases and increases alternately during the same incubation period. Ciliated protozoa numbers reached a peak at day 2–3 (8.7 × 10^3^ ciliate mL^−1^ in experiments carried out with *E. coli* and 5.5 × 10^4^ ciliate mL^−1^ in those with *S. marcescens*) that was maintained until day 4, when tagged cells had already decreased. Somatic coliphage populations were initially present, but they were undetectable after a few days of the study.

### Fate of *E. coli* and *S. marcescens* in laboratory scale experiments

**Figure 3:**
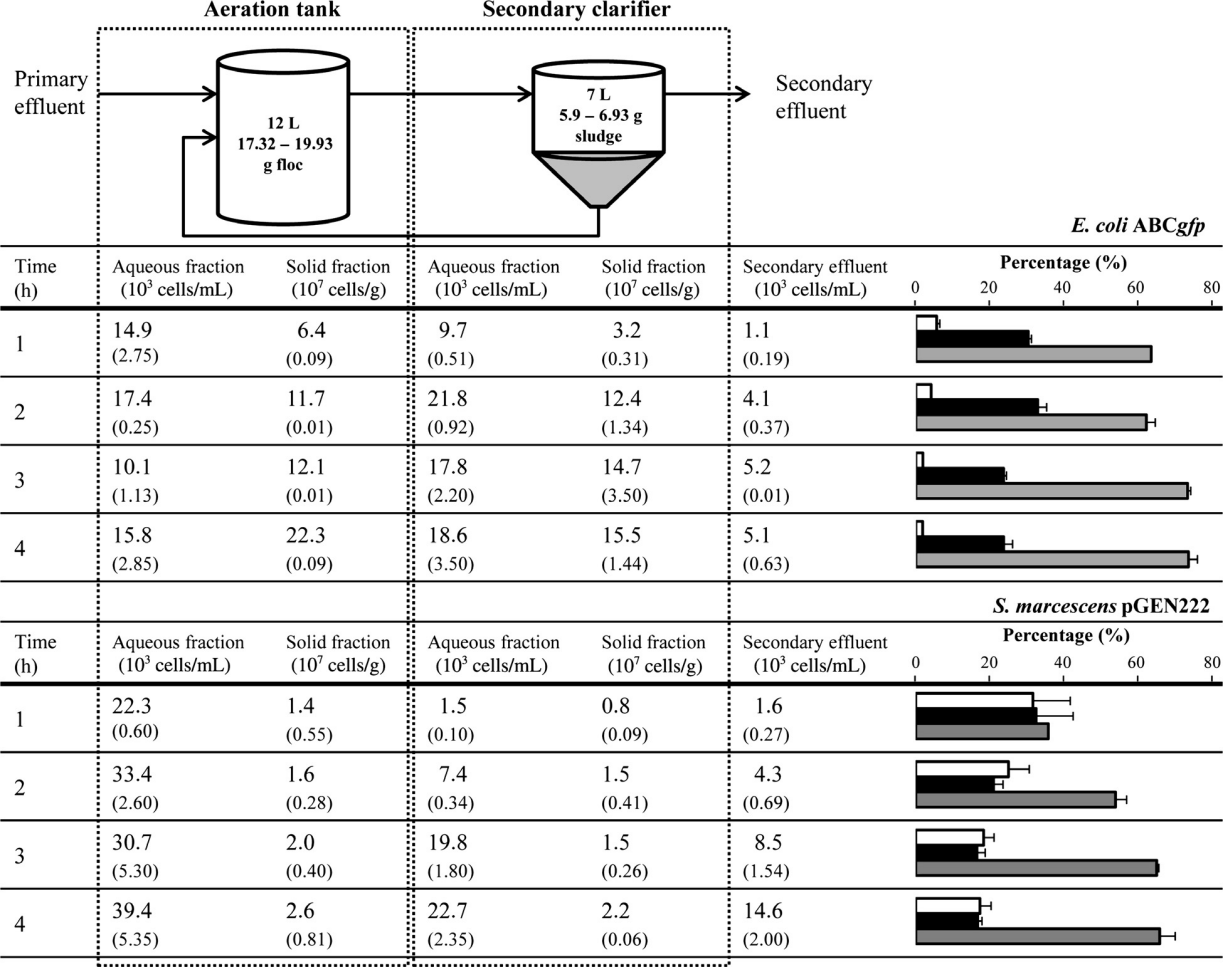
Distribution of *gfp*-tagged bacteria during treatment in the laboratory scale-activated sludge unit (ASU) after continuous inoculation over 4 h. The table presents the numbers (mean and standard deviation) of *Escherichia coli* ABC*gfp* or *Serratia marcescens* pGEN222 in the aeration tank, secondary clarifier and effluent. Numbers of *gfp*-tagged bacteria were obtained in unstained subsamples by epifluorescence microscopy. The graphs on the right represent the percentage of inoculated cells found in the different phases – aqueous (white) and solid (black) – and the bacteria that disappeared from the system (gray), calculated as the difference between the inoculated cells and those found in the ASU.

Figure3 shows the concentrations of cells in the secondary effluent and in the different fractions sampled from the ASU, which were obtained after its inoculation with tagged cells. This figure also presents the distribution of total tagged cells remaining in the WWTP and the percentage of cells that disappeared from the system in relation to those introduced over the course of the experiment. From these data, it is apparent that *E. coli* ABC*gfp* and *S. marcescens* pGEN222 exhibited important differences when they were separately introduced in the ASU.

In these experiments, when the ASU was inoculated with *E. coli* ABC*gfp*, the first hour of incubation resulted in lower values, but then the counts of this bacterium reached a steady state with a cell density ∼1–2 × 10^4^ cells mL^−1^ in aqueous fractions and 1–2 × 10^8^ cells g^−1^ in solid fractions of the ASU. In the secondary effluent, the cell density reached 5.1 × 10^3^ cells mL^−1^. *Escherichia coli* cells remaining in the system were mainly distributed into the solid fractions, flocs and sludge. Indeed, at the end of the experiments, the numbers of cells embedded in solids were ∼13 times higher than those free-living in water (the 23.69% of the inoculated cells were counted in the solid fractions, while only the 1.82% remained in the water).

For *S. marcescens* pGEN222, cells discharged into the secondary effluent increased from 1.6 × 10^3^ to 1.46 × 10^4^ *S. marcescens* mL^−1^ over the period of time during which the ASU was fed with this bacterium. In a similar way, there was a gradual increase in the number of tagged *S. marcescens* recovered from the different fractions of the ASU during these 4 h. In contrast to *E. coli*, there were differences between the numbers of cells counted in the aeration tank and those recovered from the secondary clarifier, where the cellular densities were lower (*P* ≤ 0.05). At the beginning of the experiments, 2.23 × 10^4^ *S. marcescens* mL^−1^ were present in the aqueous fraction of the aeration tank and only 1.5 × 10^3^ *S. marcescens* mL^−1^ in the secondary clarifier. High concentrations of cells were detected in solid phases, but these were ∼1 log lower than those detected in experiments inoculated with *E. coli*. As for *E. coli*, introduced cells tended to disappear (70%) from the system; however, the percentage of the remaining *S. marcescens* population was similar in the aqueous and solid fractions.

### Fate of wild *E. coli* cells in laboratory scale experiments

**Figure 4:**
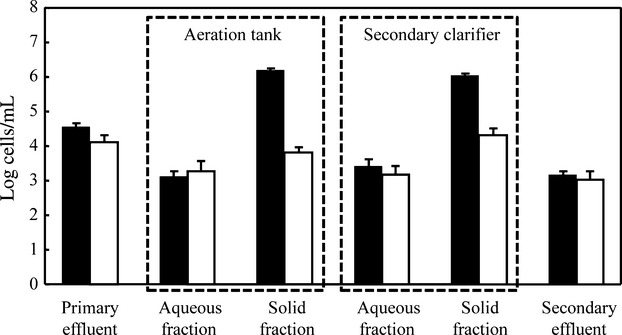
Numbers (and standard deviation) of wild *Escherichia coli* cells detected by CARD-FISH (closed bars) and MPN (open bars) methods in primary effluent, the aeration tank (aqueous and solid fractions), the secondary clarifier (aqueous and solid fractions) and the secondary effluent in the laboratory scale-activated sludge unit (ASU) not inoculated with *gfp*-tagged bacteria. Data are averages of three experiments. CARD-FISH, catalyzed reporter deposition fluorescence in situ hybridization; MPN, most probable number.

Figure4 shows the results of wild *E. coli* enumeration in the ASU, obtained from experiments carried out with uninoculated wastewater. The most remarkable result to emerge from this figure is the great difference between the counts performed for the aqueous and solid fractions (*P* ≤ 0.05). While only slight differences were detected between wild *E. coli* counts obtained by CARD-FISH and MPN in the aqueous fractions (*P* > 0.05), CARD-FISH counts were 1.5–2 logs higher in flocs and sludge (solid fraction) (*P* ≤ 0.05), highlighting the relevance of the unculturable population in solids. In addition, and similar to results previously shown, CARD-FISH counts from the solid fractions were 1.5–2.5 logs higher than those obtained from aqueous fractions (*P* ≤ 0.05).

## Discussion

The present study was designed to determine how the activated sludge process affects the removal of two related bacteria: *E. coli* and *S. marcescens*. Given the complex community structures and functioning of the WWTPs, both bacteria were modified to express green fluorescent protein before starting the experiments. Although Lowder and Oliver (2001) indicated that permanence under stressful conditions can induce loss of the *gfp* gene or stop the production of GFP, the GFP-tagged bacteria used in this study maintained fluorescence for a long period of time, and *E. coli* ABC_*gfp*_ and *S. marcescens* pGEN222 were completely distinguishable from the autochthonous bacteria. These data agree with those obtained previously in wastewater (Eberl et al. 1997; Na et al. 2006).

Experiments in an ASU and in batch systems were run in parallel to facilitate study of the activated sludge process. ASU experiments permitted us to determine the reduction efficiency for each strain by comparing secondary effluents with the total inoculated population. At first sight, the removal of both bacteria was similar. In fact, after 4 h, ∼70% of the inoculated tagged cells had disappeared from the ASU (Fig.3), they were counted neither in the solids nor in the water and only a small fraction was discharged in the secondary effluent. However, as discussed below, a more detailed analysis of ASU function revealed the differential behavior of these strains during the wastewater treatment process.

Bacterial removal in the activated sludge process has mainly been attributed to adsorption to sludge and interactions with the WWTP microbial community; these mechanisms have been reported in the literature (Atlas and Bartha 1997). Recently, the induction of the VBNC state by stressful conditions during wastewater treatment has also been highlighted (van Frankenhuyzen et al. 2011).

Eberl et al. (1997) highlighted the importance of adhesion to solid fractions to remove cells from wastewater during treatment. For *E. coli*, almost 95% of cells remaining in the ASU were present in flocs and sludge, and therefore adhesion was presumed to be a relevant factor implicated in the removal of this bacterium. In contrast, the remaining *S. marcescens* cells were equally distributed in the liquid and solid fractions.

Zita and Hermansson (1997) related CSH and cell surface charge to the attachment of bacteria to sludge flocs, indicating that hydrophobic bacteria in general attach in higher numbers to sludge flocs compared to hydrophilic cells. However, bacteria often change their hydrophobicity rapidly in response to nutrient fluctuations (Kjelleberg and Hermansson 1984), the presence of fimbriae (Jones et al. 1996) or the ionic strength and ionic valence of the aqueous phase (Jones et al. 1996).

In our work, both the *E. coli* and *S. marcescens* strains exhibited similar CSH values (no significant differences) and could be considered moderately hydrophobic. However, their distribution in the solid and liquid fractions was clearly different. Therefore, CSH values do not appear to be appropriate to predict the relative adhesion of bacteria in a heterogeneous system as was previously proposed (Olofsson et al. 1998).

Nevertheless, when the ability to form biofilm was analyzed, the large presence of *E. coli* in the solid fractions (flocs and sludge) coincides with its classification as a strongly adherent strain, in contrast with the results obtained for *S. marcescens*, a moderately adherent strain. Thus, the ability to form a biofilm appears to determine how bacteria persist in the system.

In the different habitats, allochthonous populations interact with natural microbial communities. Bacteriophages and protozoa have been identified as responsible for the elimination of microorganisms from the system in the activated sludge process (Werker et al. 2007; Ni et al. 2010; Shapiro et al. 2010). Shapiro et al. (2010) and Whitey et al. (2005) indicated that the dense biomass in wastewater treatment bioreactors, maintained under homogenized, relatively stable conditions, makes these systems ideal hunting grounds for bacteriophages. In this study, although somatic coliphages were detected in the influent of the WWTP, direct quantification of their effect upon *E. coli* and *S. marcescens* during treatment in the ASU was difficult. However, in experiments run in batch systems, the presence of bacteriophages did not affect the population density in wastewater.

Our results in the batch systems indicate that the decrease in population density appeared to be related to the presence of protozoa since in absence of this predator community no decreases were observed in bacterial populations. These results agree with those published by other authors (Ratsak et al. 1996; Wen et al. 2009), who have highlighted the importance of protozoa in the activated sludge process, given that they feed on pathogenic and fecal bacteria. The maintenance of the ratio heterotrophic bacteria/protozoa and the decrease of the ratio allochthonous bacteria/protozoa during the experimentation period corroborated this hypothesis. Studies indicate that a minimum population size may exist in which bacteria can be maintained in the presence of predators. If the amount of bacteria is below this level (in our study, 10^3^–10^4^ bacteria mL^−1^), decline due to predation is little or none (Stevik et al. 2004). Besides, following the addition of tagged strains to the batch systems, these bacteria became alternative prey for protozoa (Arana et al. 2003). The wastewater bacteria behaved as prey escaping predation and maintained their population density, while *E. coli* ABC*gfp* or *S. marcescens* pGEN222 were preyed upon.

Korajkic et al. (2013) and Wanjugi and Harwood (2013) have remarked that competition for nutrients with autochthonous bacteria also affects the survival of fecal indicator bacteria such as *E. coli*. Indeed, in freshwater sediments, competition with autochthonous bacteria influenced *E. coli* survival more than predation (Korajkic et al. 2013). However, other authors (Abhirosh et al. 2009) have established that competing autochthonous bacterial species have no effect on allochthonous bacteria. In this study, this relationship was not studied directly, and therefore we cannot rule out its importance in the control of the *E. coli* and *S. marcescens* populations. Regardless, this does not call into question the effective role of bacterivorous protozoa on fecal bacteria removal.

Recently, differences in bacterial counts performed by culture-dependent and culture-independent methods have questioned the true ability of WWTPs to remove microorganisms. Muela et al. (2011) detected a variable fraction of unculturable active cells in wastewater samples and indicated that this fraction could include the so-called viable but nonculturable (VBNC) bacteria (Arana and Barcina 2008; Oliver 2010). Moreover, in the last few years, some studies (Sawaya et al. 2008; van Frankenhuyzen et al. 2011) have focused on the risks of inducing this state during wastewater treatment. Comparison of CARD-FISH and MPN counts during treatment allows discarding the presence of VBNC *E. coli* cells in effluents from ASU experiments. However, this comparison also highlights the importance of the VBNC cells after adhesion of *E. coli* to flocs and sludge. These data suggest that the stress level in solid and liquid fractions was different and, therefore, the mechanisms involved in bacteria removal could vary in these fractions. Moreover, the presence of VBNC populations in flocs and sludge could be somehow related with competition with native bacteria, which are present in high densities (Orruño et al. 2013) in this environment.

In brief, different biological factors are implied in bacterial removal from the wastewater, namely predation by bacterivorous protozoa, bacteriophage-mediated lysis, competition with native bacteria and spontaneous cell death. From batch experiments, it can be inferred that spontaneous and bacteriophage-mediated lysis had not a relevant role in this process. The importance of competition with other bacteria is more difficult to quantify but CARD-FISH results suggested that this mechanism could mainly affect the adhered cells and it would be related with the loss of culturability in these populations. Therefore, predation appears to be the primary mechanism involved in bacterial removal during the activated sludge process. However, a representative number of bacteria (about 30%) persisted in the system. These remaining populations can be found as free-living or embedded cells, and their distribution into liquid or solid fractions varied depending on the bacterium tested; this variation even existed between closely related bacteria such as *E. coli* and *S. marcescens*. Consequently, the treatment plant itself should be taken into account since different systems exist, involving liquid or solid fractions in varying proportions and used in different ways, which could affect the bacterial removal process. Additionally, at least for *E. coli*, VBNC cells constituted an important part of the bacterial population that is adhered to solid fractions, resulting in nonculturability becoming a factor to consider during wastewater treatment. These facts emphasize the need for reliable quantitative and qualitative analysis tools for the evaluation of pathogenic microbial composition in sludge. In a previous work (Muela et al. 2011), we have concluded that microbiological parameters are essential to monitor the correct WWTP operation. In this study, we consider that it is necessary to review the real value of bacterial indicators for the control of the wastewater treatment process, as their removal patterns are not useful for predicting the behavior of other bacteria. Moreover, evidence of the existence of nonculturable but potentially active cells in sludge could represent an undefined risk to public health and ecosystem functions when considering sludge recycling. This activity could contribute to the spread of pathogens, which in the VBNC state escape the controls, and the dissemination of antibiotic resistances, among other hazards.
